# High-performance work systems and individual performance: a longitudinal study of the differential roles of happiness and health well-being

**DOI:** 10.3389/fpsyg.2023.1261564

**Published:** 2024-01-17

**Authors:** Lulu Shi, Marc Van Veldhoven, Dorien Kooij, Karina Van De Voorde, Maria Karanika-Murray

**Affiliations:** ^1^School of Management, Xi’an Jiaotong University, Xi’an, Shaanxi, China; ^2^Department Human Resource Studies, Tilburg University, Tilburg, Netherlands; ^3^School of Business, College of Social Sciences, Arts, and Humanities, University of Leicester, Leicester, United Kingdom

**Keywords:** employee well-being, perceived HPWS, happiness well-being, health well-being, individual performance, longitudinal research

## Abstract

As a part of the growing strand of employee-centered HRM research, employee well-being is suggested to be a key mechanism that may help to explain the relationship between HRM and performance. To investigate how an employee’s well-being mediates the HRM-performance relationship, we distinguish between two types of well-being identified in prior work, happiness well-being and health well-being, and present arguments for differences in their effects on individual performance. Building on Job Demands-Resources (JDR) theory, we propose that happiness well-being positively mediates the relationship between perceived High-Performance Work Systems (HPWS) and individual task performance, while health well-being negatively mediates this focal relationship. Thus, happiness well-being fits the “mutual gains” perspective. In contrast, health well-being fits the “conflicting outcomes” perspective, and thus may be harmed by the HPWS to enhance the performance. We find partial support for our arguments in an analysis of longitudinal survey data of 420 participants spanning a total of four waves of data collection.

## Introduction

Although there has been some work that looks at how the High-Performance Work System (HPWS) shapes employee-centered outcomes (Malik and Lenka, 2019), this remains an underdeveloped area of study (Jiang et al., 2013). Prior research has emphasized well-being as an important employee-centered outcome and it plays an important role in affecting employee’s performance (Van De Voorde and Beijer, 2015; Peccei and Van De Voorde, 2019). Often, HPWS practices create a “win-win” situation where employee well-being and performance are both strengthened (Peccei et al., 2013). However, well-being may be harmed in some cases due to HPWS practices that aim to optimize employees’ performance (Ogbonnaya et al., 2017). Thus, although scholars agree that employee well-being is a critical factor in explaining the relationship between perceived HPWS and individual performance, contradictory findings point to the need for further research.

One reason for these contradictory findings could be that in prior work different types of well-being have been studied (Grant et al., 2007; Alfes et al., 2012; Jiang et al., 2012; Devonish, 2013; Jackson et al., 2014). Peccei and Van De Voorde (2019) noted that multiple definitions, conceptualizations, and dimensions have emerged across studies of employee well-being. While a number of studies have focused on affective commitment and job satisfaction (Choi, 2014; García-Chas et al., 2014; Zhang et al., 2016), others have focused on job stress and anxiety (Alfes et al., 2012; Jensen et al., 2013). Rather than attempting to converge on a single definition of well-being, scholars have begun to investigate different dimensions of well-being, and have recognized that employees can for example feel satisfied while at the same time feel exhausted (e.g., Grant et al., 2007; Peccei and Van De Voorde, 2019). Important questions concerning well-being trade-offs have, however, received little or no attention in extant HRM research (Peccei and Van De Voorde, 2019). Attending to different aspects of well-being can provide greater theoretical clarity to this issue.

In this study, we investigate how different types of employee well-being mediate the relationship between perceived HPWS and individual performance. By employee well-being, we refer to “the overall quality of an employee’s experience and functioning at work” (Warr, 1990; Grant et al., 2007). Specifically, we consider two types of well-being: happiness well-being and health well-being (Page and Vella-Brodrick, 2009). Building on research into the circumplex model (Russell, 2003), there is a distinction between the dimensions of happiness and health in work-related well-being. Happiness well-being describes “subjective experiences of pleasure or the balance of positive and negative feelings and thoughts in employees’ judgments” (Grant et al., 2007; Bakker and Oerlemans, 2011), whereas health well-being refers to “employees’ physiological and psychological aspects of health at work, including job-related stress, anxiety, exhaustion, and burnout” (Warr, 1990; Danna and Griffin, 1999; Spreitzer et al., 2005).

We propose that happiness well-being and health well-being mediate the relationship between employees’ perceived HPWS and individual performance, but also that their effects are different. Prior research has argued that while HRM practices are beneficial for happiness well-being, these HRM practices tend to damage health well-being due to increased workload, stress, and strain (Alfes et al., 2012; Van De Voorde et al., 2012; Guerci et al., 2022). Because trade-offs exist between different types of employee well-being arising from HPWS practices, it can be expected that happiness well-being fits the mutual gains perspective and is congruent with employee performance, while health well-being fits the conflict outcomes perspective and is often harmed when organizations pursue higher employee performance (Van De Voorde et al., 2012; Peccei et al., 2013; Violetta and Heidi, 2018). Specifically, the mutual gains perspective holds that HRM has positive outcomes for both the employers (in terms of performance) and employees (in terms of well-being) (Van De Voorde et al., 2012; Peccei et al., 2013). However, the conflicting outcomes perspective holds that HRM practices improve performance but do not necessarily benefit employees (Van De Voorde et al., 2012; Peccei et al., 2013).

Our study extends prior research into the role of employee well-being in the HPWS-performance relationship (e.g., Hauff et al., 2014; Ho and Kuvaas, 2019; Ogbonnaya and Messersmith, 2019; Liu et al., 2020) by taking a multidimensional view of well-being and developing novel arguments of the different roles that happiness well-being and health well-being play in the HPWS-performance chain. Therefore, we extend prior research into the HRM-performance chain by providing more subtle mediating effects of employee well-being. Besides, our study clarifies the well-being trade-offs in the HRM-performance relationship (Grant et al., 2007; Peccei and Van De Voorde, 2019), whereby happiness improves, but health well-being decreases as a result of HPWS, in turn, leading to improved performance. Lastly, we adopt a longitudinal perspective, assuming that HRM practices need time to materialize. This enables us to provide unique insights into HRM-performance research by adequately capturing the dynamic nature of the proposed mediational processes.

## Theoretical background and hypotheses

### The conceptual framework

Previous researchers have argued that HRM practices are interrelated and should be examined in bundles rather than in isolation to encourage desirable performance outcomes (Macky and Boxall, 2007; Takeuchi, 2009). Thus, the field of HRM research is filled with numerous models, each advocating a distinct set of practices aimed at addressing specific relationships or objectives (Butler et al., 2004). For example, high involvement work systems focus on work organization, high commitment management addresses employee relations, while HPWS combines AMO-enhancing practices that encompass both employee relationships and work organization (Han et al., 2020).

The HPWS is defined as “range of innovative human resource practices and work design processes that, when used in certain combinations or bundles are mutually reinforcing and produce synergistic effects” (Ubeda-Garcıa et al., 2018, p. 398). In the late 1990s, the AMO framework experienced a renaissance, leading to the emergence of support for categorizing HR practices into bundles that enhance ability, motivation, and opportunity (Appelbaum et al., 2000; Katou and Budhwar, 2010). While numerous studies have established direct links between individual HR practices and a wide range of targeted HR outcomes (Jiang et al., 2012), there are also studies indicating that these domains have overlapping properties, meaning that isolating their effects can be difficult (Khoreva and Wechtler, 2018). Only when the HR practices are used coherently, may their actual impact on outcomes be fully explored. As indicated by Delery (1998) the “internal fit” of HRM practices can induce complementary effects when used together in a coherent system. The rationale behind this shift is that if the system is considered a strategic asset, it should be evaluated as an integrated whole rather than a collection of isolated parts (Sun et al., 2007). Based on this logic, HPWS can be seen as a practical implementation of the AMO model. Specifically, HPWS can be grouped as three bundles of HR practices that are strategically designed to optimize employee performance by enhancing employees’ ability, motivation, and opportunity to contribute (Delery and Shaw, 2001; Harrell-Cook and Mahoney, 2001; Lepak et al., 2006).

According to the AMO model, employee performance is a function of three essential components: ability, motivation, and opportunity to perform (Boxall et al., 2008; Jiang et al., 2012). Ability-enhancing HRM practices, such as training, provide knowledge and skills to employees, thereby fostering individual performance. In addition to the quantity of knowledge and skills conveyed, quality is also essential, while effective training can inspire employees to improve (Jiang et al., 2012; Guest et al., 2013). Motivation is defined as an individual’s direction, intensity, and duration of effort (Campbell et al., 1993). Whereas ability-enhancing HRM practices provide the capabilities for employees to perform, motivation-enhancing practices deal with the extent to which employees are willing to use these abilities (Liao et al., 2009). HR practices, such as performance feedback, may help employees perceive their work as meaningful and important, and further motivate them to perform at their best (Seibert et al., 2004). Opportunity-enhancing practices, such as flexible job design, information sharing, and autonomous work, enhance the opportunity for employees to contribute (Boxall and Macky, 2009). A feeling that one can contribute to their organization can enable employees’ proactivity. Stimulated by the aforementioned HR practices, employees’ ability, motivation, and opportunities will translate into better individual performance (Liao et al., 2009; Kuvaas and Dysvik, 2010).

**FIGURE 1:**
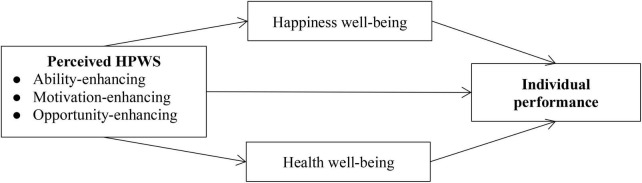
Conceptual model.

Drawing on these perspectives, we develop a conceptual framework to examine the effects of perceived HPWS on individual performance (Figure 1). Specifically, we argue that perceived HPWS positively affect individual performance. Further, we suggest that happiness and health well-being offer different mechanisms through which perceived HPWS enhance individual performance.

### Impact of perceived HPWS on individual performance

The link between employees’ perceived HPWS and individual performance can be explained by the Ability-Motivation-Opportunity (AMO) model (Appelbaum et al., 2000). According to the AMO model, employee performance is a function of three essential components: ability, motivation, and opportunity to perform (Boxall et al., 2008; Jiang et al., 2012). Based on this logic, systems of HPWS practices designed to maximize employee performance can be viewed as a composition of three bundles of HRM practices intended to enhance employee skills, motivation, and opportunity to contribute, respectively (Delery and Shaw, 2001; Harrell-Cook and Mahoney, 2001; Lepak et al., 2006).

Ability-enhancing HPWS practices, such as training, provide knowledge and skills to employees, thereby fostering individual performance. In addition to the quantity of knowledge and skills conveyed, quality is also essential; effective training can inspire employees to improve (Jiang et al., 2012; Guest et al., 2013). Motivation is defined as an individual’s direction, intensity, and duration of effort (Campbell et al., 1993). Whereas ability-enhancing HPWS practices provide the capabilities for employees to perform, motivation-enhancing practices deal with the extent to which employees are willing to use these abilities (Liao et al., 2009). Extrinsically, HPWS practices, such as direct compensation, benefits, and promoting opportunities, elicit discretionary effort and improve employee productivity (Liao et al., 2009). Intrinsically, HPWS practices, such as performance feedback, may help employees perceive their work as meaningful and important, further motivating them to perform (Seibert et al., 2004). Opportunity-enhancing practices, such as flexible job design, information sharing, and autonomous work, enhance the opportunity for employees to contribute (Boxall and Macky, 2009). When employees feel they can contribute to the organization, this can enable employees’ proactive work. Stimulated by the aforementioned HPWS practices, employees’ ability, motivation, and performance opportunities will translate into better individual performance (Liao et al., 2009; Kuvaas and Dysvik, 2010). Summarizing these established insights, we expect a positive relationship between perceptions of HPWS and individual performance. Therefore, we formulate our first hypothesis as follows:

*Hypothesis 1:* Perceived HPWS will have lagged positive effects on individual performance.

### HRM practices, well-being, and individual performance

In this study, we investigate how different types of employee well-being, which are happiness well-being and health well-being, mediate the HPWS-Performance relationship. Happiness well-being is employees’ positive experiences about their work situations (Grant et al., 2007). According to the circumplex model, employee happiness is considered a positive type of work-related well-being (Russell, 1980, 2003). One key aspect of happiness well-being is job satisfaction, which has been adopted in most employee well-being studies (Bakker and Oerlemans, 2011; Peccei and Van De Voorde, 2019). Health well-being describes employees’ physiological and psychological aspects of health at work, including job-related stress, anxiety, exhaustion, and burnout (Danna and Griffin, 1999). Based on Russell (1980, 2003) circumplex model, health well-being emphasizes the negative aspects of work-related well-being and mainly addresses mental health issues, such as anxiety, fatigue, and stress (Warr, 1990; Spreitzer et al., 2005). By studying employee happiness well-being and health well-being, we respond to recent literature calls to clarify the well-being trade-offs in the HPWS-performance relationship (Grant et al., 2007; Peccei and Van De Voorde, 2019).

It can be expected that happiness well-being fits a mutual gains perspective and is congruent with employee performance, while health well-being fits a conflicting outcomes perspective and is often harmed when organizations pursue higher employee performance (Van De Voorde et al., 2012; Peccei et al., 2013; Violetta and Heidi, 2018). Specifically, the mutual gains perspective holds that HPWS has positive outcomes for both the employers (in terms of performance) and employees (in terms of well-being) (Van De Voorde et al., 2012; Peccei et al., 2013). However, the conflicting outcomes perspective holds that HPWS practices improve performance but do not necessarily benefit employees (Van De Voorde et al., 2012; Peccei et al., 2013).

To understand such differential well-being mediation mechanisms through which HRM perceptions influence individual performance, one of the classical organizational behavior theories in explaining employee attitude and behavior is the job demands and resources (JD-R) model (Demerouti et al., 2001). In this study, the JD-R model offers a comprehensive framework for comprehending the interplay among perceived HPWS, employee well-being, and individual performance. The JD-R model posits that job characteristics can be categorized into two main components: resources and demands (Demerouti et al., 2001). Job resources are characterized by their supportive nature, which brings benefits to employees (Schaufeli and Bakker, 2004). In contrast, job demands lead to the depletion of resources enjoyed by employees and can result in adverse outcomes, including heightened anxiety and mental health issues (LePine et al., 2016). In the current study, we explicitly examine two differential processes: (1) following a mutual gains perspective, we explore how perceptions of HPWS that provide job resources may positively affect happiness well-being, in turn enhancing employee performance, and (2) following a conflicting outcomes perspective, we study how perceptions of HPWS that result in job demands may improve individual performance via exerting stronger pressure on employees.

In the JD-R model, job resources refer to everything that helps employees achieve their goals, including work autonomy, performance feedback, social support, and superior guidance (Bakker and Leiter, 2010). For employees, the practices in HPWS, such as training, job autonomy, salary fairness, promotion channels, support them to acquire promotion, increase their salary, and realize their value. Therefore, in this research, we select perceived organizational support as the “resource path” through which HPWS affects the well-being of the employee positively.

Consistent with the motivational process proposed by the JD-R research, job resources provided by HR practices can lead to increased happiness well-being (Crawford et al., 2010; Snape and Redman, 2010; Christian et al., 2011). Employees who perceive organizational commitment toward supporting their job performance, motivating their efforts, and appreciating their contributions experience an increase in their happiness well-being (Allen et al., 2003; Nishii et al., 2008). For example, a positive relationship between training and job satisfaction has been found in previous studies (Sahinidis and Bouris, 2008). Researchers have also argued that when employees perceive a high level of organizational support, they may view it as a long-term investment in them and feel satisfied with their jobs, and therefore engage in desirable work-related behavior (Lambert, 2000; Shaw et al., 2009).

We thus build on both these streams of research to propose that perceived HPWS has a positive effect on individual performance by enhancing their happiness well-being. Overall, we expect that happiness well-being positively mediates the relationship between perceived HPWS and individual performance. Since HPWS can directly contribute to increased performance by enhancing employees’ abilities, motivations, and opportunities to perform, we expect happiness well-being to partially mediate the HPWS-performance relationship. A hypothesis is thus formulated as:

*Hypothesis 2a:* Perceived HPWS is positively related to happiness well-being, and happiness well-being, in turn, is positively related to individual performance, such that happiness well-being positively mediates the effect of employees perceived HPWS on individual performance.

Consistent with the health impairment process proposed by the JD-R model, chronic job demands caused by intensive HPWS practices may exhaust employees’ mental resources and therefore result in health problems (Demerouti et al., 2001). As shown in Kroon et al. (2009) study of HR managers and employees in various organizations, employees’ perceptions of job demands increased as their perceptions of HPWS utilization increased. Work redesign practices focused on enriching assigned tasks can increase job satisfaction but also undermine employee health (Grant et al., 2007). Enriched jobs provide more opportunities to stretch the skills necessary to complete complex tasks. These opportunities may be perceived as responsibilities and burden decision-making, therefore leading to increased perceived job demands (Campion and McClelland, 1991, 1993). For example, employees in a large financial service company reported increased physical strain, overload, and health complaints when they were given more responsibilities (Campion and McClelland, 1993). Similarly, too much training can also take valuable time away from task execution, also adding to time pressures (Grant et al., 2007; Warr, 2007). Therefore, HPWS may be perceived as increased job demands and result in additional enhanced stress (Topcic et al., 2016).

To maintain or increase profitability, organizations are under constant pressure to implement progressive HPWS to achieve performance goals (Peccei and Van De Voorde, 2019). In particular, progressive HR practices may make employees feel exploited at work (Truss et al., 1997) and therefore harm rather than benefit employee well-being. For instance, HPWS practices such as performance management, training, and job design, can result in a greater intensification of work and more systemic exploitation of employees, all of which can be harmful to their well-being (Peccei, 2004). With these HPWS practices, employees have to work harder and be under greater pressure, which can enhance their job performance (Peccei et al., 2013). As indicated by Kroon et al. (2009: 512), “Although employees may value the incentives offered to them through HPWS practices, the message that the system signals to the employees are one of increasingly higher performance, and that it is the company which ultimately benefits from the individuals’ extra effort” (Legge, 1995). To protect resources they hold in organizations, employees must invest time, energy, and other personal resources to prevent the loss of resources, such as salary and promotion opportunities. The gap between resources the employees need to invest and resources they hold will cause increased work stress immediately (Hobfoll et al., 2018).

We thus build on both these streams of findings to propose that HPWS, as perceived by the individuals, have a positive effect on individual performance but can harm health well-being. Overall, we expect that health well-being negatively mediates the relationship between perceived HPWS and individual performance. Since HPWS can directly contribute to increased individual performance by building the abilities, motivations, and opportunities to perform, we expect health well-being to partially mediate the HPWS-performance chain. The hypothesis is thus formulated as:

*Hypothesis 2b:* Perceived HPWS are negatively related to health well-being, and health well-being is, in turn, negatively related to individual performance, such that health well-being negatively mediates the effect of HPWS on individual performance.

## Methodology

### Participants and procedure

Participants in this study were individuals enrolled in a large-scale project between 2013 and 2016 in the UK with private and public organizations and aimed to study the factors that impact health, well-being, and work outcomes. After consultation with the organization’s management and employee representatives, a longitudinal self-report online survey was adopted as the data collection method. The participants were informed about the research purpose, anonymity, and confidentiality of data, and could withdraw from the study at any time. Incentives were offered in a prize draw for a media player.

The questionnaire was distributed every 3 months, and four waves of data were collected during one calendar year. All items were collected during each wave of data collection. A longitudinal approach to data collection aligns to the temporal nature of theory development (Ployhart and Vandenberg, 2010). There are two reasons for the adoption of the longitudinal research design. First, it allowed for exposure to HRM practices to produce effects on individuals’ behaviors and affective outcomes (Lapré and Nembhard, 2020). Second, using four measurement waves is needed to adequately test for mediation. This also meets the minimum number of three repeated measures for enhanced reliability in HR research (Ployhart and Vandenberg, 2010) and allows us to capture the actual effects of the focal variables by controlling the influences of the same variable in previous waves (Little et al., 2007).

The participants in this study were employees from 16 organizations in various industries, including manufacturing and technology, construction, finance, retail, emergency services, education, and local government. Consequently, their occupations and roles were highly diverse and included administrative staff, senior managers, middle managers, professional workers, manual workers, technical workers, and service staff. With the participants coming from variable industries and job positions, we can expect variation in HRM perceptions. A total of 5,086 questionnaires were sent out in each wave of data collection. The average response rate for each wave were: 36, 43, 34, and 30%. In a survey methods study, Kaplowitz et al. (2004) found that response rates for an online survey through email are about 21%, lower than the lowest response rate in our research. Therefore, the response rate in the current study is acceptable for online surveys.

After listwise deletion of missing data, measures of perceived HRM practices, employee well-being, and individual performance yielded a total of 1,680 questionnaires with complete data from all four waves generated from *N* = 420 individuals. Females comprised 61% of the study sample, mean age was *M* = 42.76 (SD = 10.48), and 46.6% of the sample were educated at secondary school level.

### Measures

#### Perceived HPWS

Research has shown differences among intended, implemented, and perceived HRM practices (Liao et al., 2009; Kehoe and Wright, 2013). Due to implementation variability and individual diversity, the links between intended or implemented HRM practices and perceived HRM practices tend to be weak (Den Hartog et al., 2012; Fu et al., 2020). Previous research has further suggested that one’s perceptions of HRM practices are more proximal to their behaviors and thus better in predicting their performance (Alfes et al., 2013; Jiang et al., 2017). Following this line of reasoning, it is sensible to focus on employees’ own perceptions of HPWS when examining employee-centered outcomes.

This measure assesses employees’ perceptions of the HPWS implemented by their respective organizations. Building on previous research (e.g., Boselie et al., 2005; Wall and Wood, 2005; Jiang et al., 2012), the perceived High-Performance Work System (HPWS) can be measured as a reflective-formative second-order construct, which comprises three first-order dimensions: ability-enhancing practices, motivation-enhancing practices, and opportunity-enhancing practices. Specifically, we tapped into perceptions concerning ability-enhancing practices by asking the participants about the use of training needs evaluations and training and career development programs (Boxall et al., 2011), given that training is likely to increase their level of knowledge and skills. We assessed participants’ perceptions of motivation-enhancing practices by asking them about the extent to which they perceived the existence of incentive systems that recognized their work (Sun et al., 2007) and granted promotions based on performance (Huselid, 1995) because such performance-based rewards and promotions are likely to enhance their motivation to perform. We assessed opportunity-enhancing practices by asking about opportunities for participation in decision-making processes (Sikora et al., 2015) and whether work efforts were appreciated by their employers (Sun et al., 2007) because employees who believe that they play an important role and that their voice counts in an organization tend to perceive greater opportunities to contribute.

#### Individual performance

We measured individual performance using Griffin et al.’s (2007) 3-item scale. One example item asked the participants about the extent to which they carried out the core parts of their job well. The questions were answered on a 5-point Likert scale, ranging from 1 = “strongly disagree” to 5 = “strongly agree.” The Cronbach’s alphas of this measure for the waves were 0.89, 0.88, 0.91, and 0.89.

#### Happiness well-being

We measured happiness well-being as job satisfaction, which was assessed by a combination of 3 questions commonly used in research (e.g., Cammann et al., 1979; Riordan et al., 2005; Gould-Williams, 2007). The participants were asked to describe whether they agreed with three statements. One example item is “In general, my job measures up to the sort of job I wanted when I took it.” Answers were captured with a 7-point Likert scale ranging from 1 = “strongly disagree” to 7 = “strongly agree.” A higher score meant a higher level of job satisfaction. Cronbach’s alphas of this measure for the four waves were 0.89, 0.89, 0.91, and 0.90.

#### Health well-being

We measured health well-being using Daniels’s (2000) anxiety-comfort scale, which was developed to measure anxiety as low pleasure and high mental arousal (based on Warr, 1990). This measure was selected because of its common representation as a component of employee health well-being (Warr, 1990; Spell and Arnold, 2007; Bergersen et al., 2010). Specifically, a high level of anxiety is accompanied by worry, tension, and inability to relax (Spell and Arnold, 2007). The respondents were asked to describe how often six adjectives applied to them at work. For instance, “Thinking of the past week, how much of the time has your job made you feel relaxed?.” Answers ranged on a 6-point scale from 0 = “never” to 5 = “all the time.” A higher score indicates lower levels of anxiety and therefore better employee mental health. Cronbach’s alphas of this measure for the four waves were 0.92, 0.92, 0.92, and 0.93.

#### Control variables

Previous studies have found that demographic variables (e.g., age, education and gender) significantly influence performance (Ng and Feldman, 2008). Therefore, we controlled for these variables in our analyses.

### Preliminary analysis

All items were measured on a 7-point Likert scale ranging from 1 (strongly disagree) to 7 (strongly agree) in the questionnaire. We then conducted exploratory factor analyses for each data wave using principal component extraction and the varimax rotation method to examine the underlying factor structure of the HPWS (Table 1). The results of the analyses revealed a three-factor solution that worked for each of the four waves of data (explained variance for the four waves was 81.05, 79.73, 82.31, and 83.17%, respectively). One item that did not load on the motivation dimension was deleted; this item was about regular formal performance evaluation, and its loading factor was only 0.14. A secondary confirmatory factor analysis was conducted next, and the results showed a good fit of the data for all four waves of data. Specifically, Chi-square fit statistics for Wave 1 secondary CFA are RMSEA (0.09), CFI (0.98), NFI (0.95), TLI (0.95), and IFI (0.98). Secondary CFA fit statistics for Wave 2 are RMSEA (0.09), CFI (0.98), NFI (0.98), TLI (0.94), and IFI (0.98). Secondary CFA fit statistics for Wave 3 are RMSEA (0.10), CFI (0.98), NFI (0.98), TLI (0.95), and IFI (0.98). Secondary CFA fit statistics for Wave 4 are RMSEA (0.10), CFI (0.98), NFI (0.97), TLI (0.93), and IFI (0.97). The CFA results provided evidence for the validity of the newly created HPWS measure. In addition, loading of all the items ranged from 0.54 and 0.99, and thus all items could be combined into three latent variables and a resulting second-order HPWS index. The measurement invariance of this HPWS-index between different waves was tested using multigroup confirmatory factor analysis. As the Chi-square difference test is significantly affected by sample size, the differences between goodness-of-fit indexes CFI and TLI (i.e., ΔCFI and ΔTLI) were used in this study to examine measurement invariance across time waves (Meade et al., 2008). As shown in Table 1, the values of ΔCFI and ΔTLI were all less than 0.01, which indicates that there was no significant difference between the two models (Cheung and Rensvold, 2002). Therefore, the measurements of HPWS perceptions are acceptable in terms of cross-time invariance.

**TABLE 1:** Exploratory factor analysis on human resource practices items and summary of scale properties.

		Estimate				SE				*T*-value				R-square				Standardized estimate			
		T1	T2	T3	T4	T1	T2	T3	T4	T1	T2	T3	T4	T1	T2	T3	T4	T1	T2	T3	T4
HPWS	Ability	1.00	1.00	1.00	1.00	–	–	–	–	–	–	–	–	0.72	0.45	0.33	0.56	0.85	0.67	0.79	0.75
	Motivation	0.39	0.60	0.53	0.61	0.07	0.09	0.08	0.08	5.67	6.52	6.63	8.05	0.32	0.29	0.67	0.35	0.57	0.54	0.58	0.59
	Opportunity	0.58	0.77	0.81	0.84	0.07	0.12	0.09	0.09	7.89	6.71	8.89	9.12	0.52	0.67	0.62	0.78	0.72	0.82	0.82	0.88
Ability-enhancing	Eliciting training needs based on performance evaluation	1.00	1.00	1.00	1.00	–	–	–	–	–	–	–	–	0.92	0.99	0.89	0.55	0.96	0.99	0.94	0.97
	Training and skills development	0.89	0.86	0.94	0.88	0.04	0.05	0.04	0.04	21.56	18.75	22.67	23.27	0.76	0.73	0.82	0.69	0.87	0.85	0.90	0.89
	Career development	0.98	0.92	0.88	0.94	0.04	0.04	0.04	0.04	22.59	21.30	21.09	23.91	0.86	0.81	0.69	0.94	0.93	0.90	0.83	0.90
Motivation-enhancing	Incentive systems that recognize employee’s work	1.00	1.00	1.00	1.00	–	–	–	–	–	–	–	–	0.35	0.52	0.43	0.77	0.59	0.72	0.66	0.74
	Promotion based on performance	1.59	1.14	1.36	1.09	0.23	0.15	0.16	0.11	7.08	7.89	8.35	9.53	0.92	0.70	0.85	0.81	0.97	0.84	0.92	0.83
Opportunity-enhancing	We are always aware of how well we are doing the job	1.00	1.00	1.00	1.00	–	–	–	–	–	–	–	–	0.51	0.45	0.62	0.57	0.71	0.67	0.79	0.76
	Negative feedback is given on a constructive way	1.07	1.07	1.01	1.12	0.08	0.09	0.06	0.07	14.22	12.13	16.27	16.41	0.64	0.56	0.64	0.72	0.80	0.75	0.80	0.85
	Work effort is appreciated	1.39	1.43	1.35	1.32	0.08	0.09	0.06	0.06	17.64	15.68	20.87	21.44	0.81	0.79	0.88	0.79	0.90	0.89	0.94	0.89
	We feel that we are listened to	1.50	1.53	1.31	1.41	0.10	0.12	0.08	0.08	15.11	13.23	17.26	17.07	0.88	0.86	0.82	0.87	0.94	0.93	0.90	0.93

Based on the secondary confirmatory factor analysis results, we created three indices for each wave to reflect a comprehensive measure of perceived HPWS. To do so, we calculated scale scores by averaging across the items of each practice dimension (ability, motivation, and opportunity) and then used these as observed variables to reflect the perceived HPWS practices, which were treated as latent variables, in the empirical models. The Cronbach’s alphas of the HPWS index measures were 0.90, 0.88, 0.90, and 0.91 for Waves 1, 2, 3, and 4, respectively. A high score on this index indicated that the participants were experiencing extensive HPWS implemented by their organizations.

As shown in Table 2, the values of ΔCFI and ΔTLI are all lower than 0.01, which indicates that the measurements of individual performance, happiness well-being and health well-being are acceptable in terms of cross-time invariance.

**TABLE 2:** Measurement invariance tests across four waves.

Variable	Model	S-Bc^2^	*df*	CFI	TLI	RMSEA	ΔCFI	ΔTLI
HRM practices perceptions	Model 1	67.12	52	0.999	0.996	0.013	—	—
	Model 2	88.32	70	0.998	0.996	0.012	−0.001	0
	Model 3	96.96	79	0.998	0.997	0.012	0	0
	Model 4	192.08	148	0.996	0.996	0.013	−0.002	−0.001
Individual performance	Model 1	9.69	4	0.998	0.995	0.029	—	—
	Model 2	12.37	7	0.998	0.997	0.021	0	0.002
	Model 3	12.57	10	0.999	0.999	0.012	0.001	0.002
	Model 4	58.01	19	0.988	0.992	0.035	−0.001	−0.007
Happiness well-being	Model 1	6.74	4	0.999	0.998	0.020	—	—
	Model 2	6.80	7	0.999	0.999	0.000	0	0.001
	Model 3	10.48	10	0.999	0.999	0.005	0	0
	Model 4	20.51	19	0.999	0.999	0.007	0	0
Health well-being	Model 1	83.76	24	0.994	0.985	0.039	—	—
	Model 2	92.10	39	0.995	0.992	0.029	0.001	0.007
	Model 3	95.71	42	0.995	0.992	0.028	0	0
	Model 4	130.71	60	0.993	0.993	0.027	−0.002	0.001

Model 1 = Unconstrained model; Model 2 = Measurement weights model; Model 3 = structural covariance model; Model 4 = Measurement residuals model.

#### Additional analysis

The direction of the HRM-performance relationship has provoked considerable discussion in the HRM literature (e.g., Wright et al., 2005; Combs et al., 2006; Piening et al., 2013). This study addresses this issue by hypothesizing and testing alternative reversed relationships between perceived HPWS and individual performance. A basic premise of the reversed relationship between perceived HPWS practices and performance is that organizations with high performance possess additional resources and thus reveal a higher willingness and more opportunities to invest in their HR systems (Boselie et al., 2005; Wright and Haggerty, 2005; Van Iddekinge et al., 2009). Following the norm of reciprocity, better individual performance can result and be aggregated into increased organizational financial performance, which creates obligations for the organization to share the benefits with its employees by increased investments in HR practices (Bowen and Ostroff, 2004; Den Hartog et al., 2012). Specifically, if an organization invests part of its financial resources in implementing more sophisticated HR practices to enhance employees’ skills, motivation, and opportunity to perform, employees’ perceptions of HPWS are expected to improve over time (Bowen and Ostroff, 2004; Den Hartog et al., 2012; Piening et al., 2013).

From the angle of employees’ perceptions, individual performance may likely influence the way they perceive and attach meaning to the organization’s HPWS (Nishii et al., 2008). When people underperform in their job, they may receive limited organizational identification. Underperforming employees receive less investments from the organization in terms of HRM compared to high performing employees. Therefore, they may perceive that the firm must not care about them and have negative perceptions about its HR practices (Nishii et al., 2008). The effect of performance on employees’ perceptions of HPWS can be strengthened over time since it takes time for the organization to have additional resources from employee performance and then invest this in HPWS (Shaw et al., 2013). Besides, employees tend to judge their working conditions based on previous experience, creating high temporal persistence in the expectations of HR practices (Chen et al., 2011). Summarizing these established insights, we expect a positive relationship between individual performance and perceived HPWS over time.

As shown in Appendix 1, individual performance in t1, t2, and t3 have no significant effect on perceived HPWS in t2 (β = 0.005, *p* = 0.471), t3 (β = −0.089, *p* = 0.152), and t4 (β = −0.118, *p* = 0.073), respectively. Overall, our findings do not support the idea of reciprocal relationships between HRM perception and individual performance.

### Controlling for common method bias and nesting of the data

The data are potentially subject to common method bias since they were all collected from individual respondents (Podsakoff et al., 2003). Although the longitudinal research design may reduce concerns about common method bias (because the well-being and individual performance are measured at different time waves), we addressed this potential problem by testing the common method variance. Specifically, we conducted Harman’s one-factor test to check for common method bias in the data (Podsakoff et al., 2003). The result shows that the first unrotated factor accounts for 37% of the covariance among the measures, and this is lower than the 40% threshold. This result provides evidence against there being a bias stemming from common method variance. In our study, we have collected data from the individual level. However, these individual participants are nested within different organizations. To truly capture the individual variability, we examined the nesting effect by conducting an ANOVA analysis on our core variables. The results, presented in Appendix 2, indicate that there were no significant differences observed among the participant organizations.

### Statistical procedure

Based on the assumption that HR interventions take time to realize, we use a longitudinal design to test the hypotheses (Birdi et al., 2008). As Huselid and Becker (1996) indicated, the effects of HR systems on performance are stronger over time than the contemporaneous association because time allows the exposure of HR practices to produce their effects on individual attitudes and behavioral outcomes. For example, in the case of individual learning, training transfer is not immediate. Rather, its effects may need to build up for some time, depending on various factors such as learning effectiveness (Lapré and Nembhard, 2020). Although it is not possible to know when exactly HPWS practices may produce a change in individual outcomes, we expect that the existence of HPWS will act as enablers and then reinforcers of individual everyday experience and behaviors (Mitchell and James, 2001). Similarly, it takes time for attitudes to translate into stimuli to exert their impact (Boxall et al., 2008; Nishii and Wright, 2008). In other words, it takes time for employees to change their behavior based on different perceptions of HPWS practices. Empirically, Volmer et al. (2011) and Simbula et al. (2011) showed that employee perceptions of their work environment have a positive effect on employee outcomes after 3 and 4 months, respectively. Thus, this study adopts a longitudinal research approach to examine the proposed relationships.

To examine hypothesized relationships, we used latent variable structural equation modeling procedures (Marrone et al., 2022). The model was assessed based on maximum-likelihood estimation method, the path coefficients were estimated and their significance were assessed with 5,000 sub-sample bootstrap draws and bias-corrected 95% confidence intervals. To evaluate model fit, we relied on Chi-square fit statistics, RMSEA (<0.08), CFI (>0.90), NFI (>0.90), TLI (>0.90), and IFI (>0.90) (Steiger, 1990; Hu and Bentler, 1999). The main focus of this research is to examine the individual variations in happiness and health well-being, and how they mediate the relationship between the perception of HPWS practices and individual performance. Thus, we first modeled the direct relationship between perceptions of HPWS practices and individual performance over time. Specifically, we modeled the cross-lagged effects of HPWS perceptions in Wave t on individual performance in Wave t + 1. To test for mediation, we included well-being (happiness and health) in Wave t + 1 as a mediator of the relationship between HPWS practice perceptions in Wave t and individual performance in Wave t + 2. To link temporal changes of independent and mediating variables more closely with the dependent variables, we included stability paths for each variable in the model.

### Model comparison and goodness of fit

We compared our hypothesized models with null models that only had stability paths between the same variables in the different waves. All the hypothesized models fit the data significantly better than their respective null models (Table 3). We also compared the models with only indirect paths (full mediation) and models with both direct and indirect paths (partial mediation). The comparison results show that full mediation models with both direct and indirect effects fit the data significantly better (Table 3).

**TABLE 3:** Model comparisons and goodness of fit statistics.

	*c* ^2^	*df*	RMSEA	CFI	NFI	TLI	IFI	Δdf	Δc^2^
**HRM perceptions → Individual performance**									
Null model	1022.57	380	0.06	0.93	0.90	0.92	0.93		
Cross-lagged model	1016.68	377	0.04	0.93	0.90	0.92	0.93		
Model comparison								3	5.89**
**HRM perceptions → Happiness well-being → Individual performance**									
Null model	2987.85	1,325	0.06	0.92	0.90	0.91	0.92		
Partial mediation model	2938.84	1,321	0.05	0.93	0.91	0.92	0.93		
Model comparison								4	49.01
Full mediation model	2932.20	1,319	0.06	0.92	0.91	0.92	0.93		
Model comparison								6	55.65**
**HRM perceptions → Health well-being → Individual performance**									
Null model	2733.85	1,329	0.05	0.93	0.88	0.92	0.93		
Partial Mediation model	2702.13	1,324	0.05	0.93	0.91	0.92	0.93		
Model comparison								5	31.72
Full mediation model	2700.89	1,323	0.06	0.93	0.90	0.92	0.93		
Model comparison								6	32.96**

*p < 0.05;

**p < 0.01.

## Results

Table 4 shows means, standard deviations, and correlations for all measures. The correlations of HPWS perceptions with employee job satisfaction and mental health are positive. As mentioned previously, higher scores on the measures meant better employee happiness well-being and health well-being.

**TABLE 4:** Descriptives and pairwise correlations among the study variables.

Variable	1	2	3	4	5	6	7	8	9	10	11	12	13	15	16	17	18
HPWS.t1	–																
HPWS.t2	0.81**	–															
HPWS.t3	0.77**	0.83**	–														
HPWS.t4	0.76**	0.82**	0.86**	–													
Individual Performance.t1	0.09	0.11*	0.12*	0.08	–												
Individual Performance.t2	0.09	0.08	0.08	0.06	0.40**	–											
Individual Performance.t3	0.11*	0.12*	0.12*	0.07	0.44**	0.54**	–										
Individual Performance.t4	0.13**	0.10	0.13**	0.14**	0.41**	0.46**	0.51**	–									
Happiness well-being.t1	0.59**	0.54**	0.54**	0.50**	0.21**	0.20**	0.19**	0.20**	–								
Happiness well-being.t2	0.49**	0.56**	0.53**	0.49**	0.13**	0.11**	0.20**	0.13**	0.77**	–							
Happiness well-being.t3	0.48**	0.51**	0.61**	0.54**	0.19**	0.19**	0.28**	0.26**	0.72**	0.74**	–						
Happiness well-being.t4	0.45**	0.48**	0.51**	0.56**	0.15**	0.19**	0.26**	0.28**	0.69**	0.72**	0.80**	–					
Health well-being.t1	0.37**	0.34**	0.37**	36**	0.20**	0.19**	0.22**	0.19**	0.48**	0.40**	0.42**	0.43**	–				
Health well-being.t2	0.34**	0.41**	0.41**	0.41**	0.10*	0.17**	0.18**	0.11*	0.41**	0.50**	0.45**	0.45**	0.60**				
Health well-being.t3	0.35**	0.39**	0.47**	0.45**	0.13*	0.11*	0.17**	0.17**	0.40**	0.43**	0.56**	0.51**	0.53**	–			
Health well-being.t4	0.30**	0.33**	0.38**	0.47**	0.02	0.11*	0.12*	0.22**	0.39**	0.44**	0.49**	0.59**	0.55**	0.63**	–		
Age	-0.02	0.018	0.015	0.07	-0.02	-0.08	-0.04	0.01	-0.03	0.13*	0.07	0.14**	-0.05	0.08	0.13*	–	
Education	-0.05	-0.01	0.02	0.01	-0.09	-0.14	-0.06	-0.13	0.01	0.04	0.05	0.02	-0.06	0.02	0.01	-0.05	–
Gender	0.021	-0.03	-0.03	0.01	-0.08	-0.14	-0.12	-0.12	-0.01	0.01	0.03	0.05	-0.03	0.01	0.05	0.14**	0.05
Mean	4.12	4.07	3.99	3.94	4.38	4.49	4.46	4.44	5.20	5.13	4.99	4.96	3.65	3.80	3.78	42.68	1.68
S.D	1.28	1.25	1.30	1.33	0.61	0.62	0.60	0.60	1.39	1.48	1.45	1.48	1.02	1.08	1.13	10.54	0.78

*N* = 420.

**Correlation is significant at the 0.01 level (1-tailed).

*Correlation is significant at the 0.05 level (1-tailed).

**FIGURE 2:**
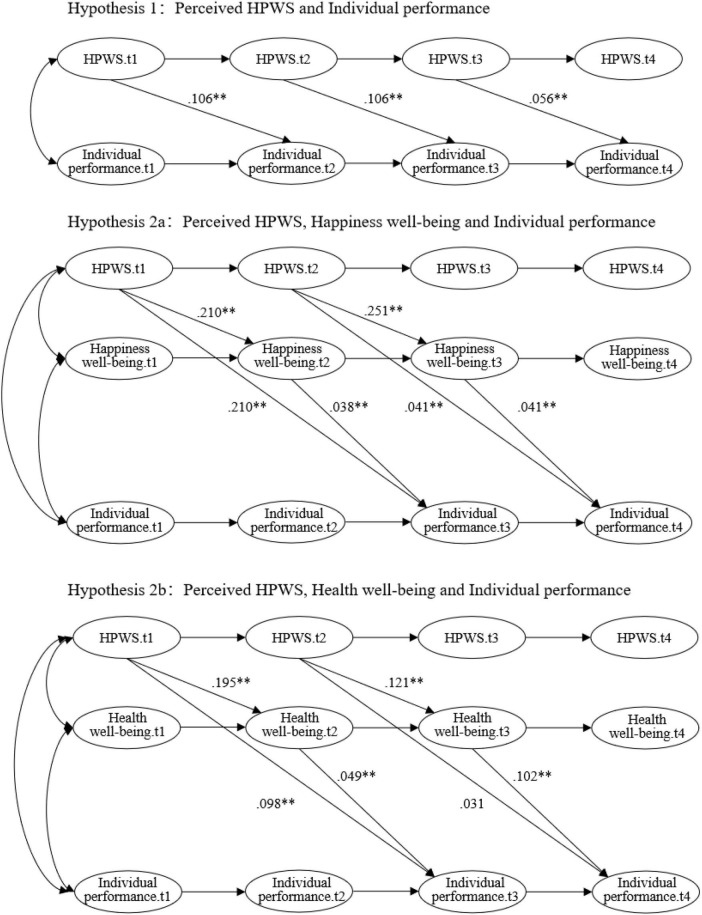
Graphical Depiction of the Standard Estimated of the Hypothesized Models. *p < 0.05; **p < 0.01.

Below, we present the results of our hypotheses testing (Figure 2). Due to the complexity of the four-wave panel data, we tested our hypotheses in various models. Hypothesis 1 posits the direct effects of perceived HPWS on individual performance. Specifically, we tested this hypothesis by examining the direct effect of perceived HPWS in Wave t on individual performance in Wave t + 2. As shown in Table 5, perceived HPWS in t1 and t2 are positively related to individual performance in t2 (β = 0.05, 95% CI [0.026, 0.129]), t3 (β = 0.045, 95% CI [0.019, 0.108]) and t4 (β = 0.024, 95% CI [0.017, 0.091]), respectively. The results in Table 5 demonstrate that perceived HPWS is positively related to individual performance. Therefore, Hypothesis 1a is supported by our research results.

**TABLE 5:** Structural Equation Modeling Analysis Results: HPWS and Individual performance.

Path	Coefficients	SE	*P*	95% CI	
				LCI	UCI
HPWS.t1 → Individual performance.t2	0.050	0.024	0.039	0.026	0.129
HPWS.t2 → Individual performance.t3	0.045	0.020	0.022	0.019	0.108
HPWS.t3 → Individual performance.t4	0.024	0.023	0.029	0.017	0.091
Age → Individual performance.t2	-0.002	0.003	0.461		
Age → Individual performance.t3	0.002	0.002	0.329		
Age → Individual performance.t4	0.001	0.003	0.814		
Education → Individual performance.t2	-0.064	0.039	0.099		
Education → Individual performance.t3	0.031	0.032	0.329		
Education → Individual performance.t4	-0.063	0.037	0.088		
Gender → Individual performance.t2	-0.129	0.063	0.039		
Gender → Individual performance.t3	-0.047	0.052	0.372		
Gender → Individual performance.t4	-0.032	0.061	0.606		

SE, standard error; 95% CI, 95% confidence interval; LCI, lower limit of confidence interval; UCI, upper limit of confidence interval.

Hypothesis 2a stated that perceived HPWS have positive effects on happiness well-being, which further positively mediates the relationship between perceived HPWS and individual performance. Specifically, we tested this hypothesis by examining the effect of perceived HPWS in Wave t on happiness well-being in Wave t + 1, and then the effect of happiness well-being in Wave t + 1 on individual performance in Wave t + 2. The results in Table 6 demonstrate that the HPWS in t1 and t2 are positively related to happiness well-being in t3 (β = 0.114, 95% CI [0.043, 0.195]) and t4 (β = 0.207, 95% CI [0.109, 0.305]), and that happiness well-being t2 and t3 are positively related to individual performance in t3 (β = 0.029, *p* < 0.05) and t4 (β = 0.038, 95% CI [0.012, 0.097]). The indirect effects were examined by the bootstrapping procedure test with a 95% confidence interval. The indirect effects of HPWS perceptions (Wave t) on individual performance (Wave t + 2) via happiness well-being (Wave t + 1) were 0.003 (95% CI [0.000, 0.020]) and 0.007 (95% CI [0.004, 0.032]). These results together support that happiness well-being partially mediate the relationship between perceived HPWS and individual performance.

**TABLE 6:** Structural Equation Modeling Analysis Results: HPWS, Happiness well-being and Individual performance.

Path	Coefficients	SE	*P*	95% CI	
				LCI	UCI
HPWS.t1 → Individual performance.t3	0.048	0.021	0.024	0.023	0.093
HPWS.t2 → Individual performance.t4	0.060	0.026	0.038	0.000	0.165
HPWS.t1 → Happiness well-being.t2	0.114	0.032	0.000	0.043	0.195
HPWS.t2 → Happiness well-being.t3	0.207	0.041	0.000	0.109	0.305
Happiness well-being.t2 → Individual performance.t3	0.029	0.037	0.042	0.012	0.097
Happiness well-being.t3 → Individual performance.t4	0.038	0.030	0.028	0.002	0.159
HPWS.t1 → Happiness well-being.t2 → Individual performance.t3	0.003	0.004	0.004	0.000	0.020
HPWS.t2 → Happiness well-being.t3 → Individual performance.t4	0.007	0.003	0.032	0.004	0.032
Age → Individual performance.t3	0.001	0.002	0.587		
Age → Individual performance.t4	0.001	0.003	0.785		
Age → Happiness well-being.t2	0.006	0.003	0.063		
Age → Happiness well-being.t3	0.004	0.004	0.329		
Education → Individual performance.t3	0.027	0.032	0.402		
Education → Individual performance.t4	-0.060	0.037	0.104		
Education → Happiness well-being.t3	0.025	0.040	0.533		
Education → Happiness well-being.t3	0.047	0.047	0.308		
Gender → Individual performance.t3	-0.057	0.052	0.270		
Gender → Individual performance.t4	-0.034	0.061	0.579		
Gender → Happiness well-being.t2	-0.012	0.064	0.848		
Gender → Happiness well-being.t3	0.100	0.075	0.186		

SE, standard error; 95% CI, 95% confidence interval; LCI, lower limit of confidence interval; UCI, upper limit of confidence interval.

Hypothesis 2b stated that perceived HPWS have negative effects on employee health, which further mediates the positive relationship between HRM perceptions and individual performance. Different from our hypothesis, HPWS at t1 and t2 are found to have positive effects on health well-being in t2 (β = 0.122, 95% CI [0.062, 0.323]) and t3 (β = 0.070, 95% CI [0.025, 0.150]) (Table 7). In addition, health well-being in t2 and t3 was found to have positive effects on individual performance in t3 (β = 0.037, 95% CI [0.002, 0.121]) and t4 (β = 0.075, 95% CI [0.027, 0.163]), respectively. The indirect effects of HRM perceptions (Wave t) on individual performance (Wave t + 2) via health well-being (Wave t + 1) were 0.008 (95% CI [0.000, 0.020]) and 0.004 (95% CI [0.000, 0.032]). No significant direct effect was found from HRM perceptions on individual performance (β = 0.047, 95% CI [−0.012, 0.087]). These results together support that health well-being significantly partially mediate the relationship between perceived HPWS and individual performance but with positive effects.

**TABLE 7:** Structural Equation Modeling Analysis Results: HPWS, Health well-being and Individual performance.

Path	Coefficient	SE	*P*	95% CI	
				LCI	UCI
HPWS.t1 → Individual performance.t3	0.047	0.025	0.063	−0.012	0.087
HPWS.t2 → Individual performance.t4	0.013	0.023	0.050	0.003	0.021
HPWS.t1 → Health well-being.t2	0.122	0.035	0.000	0.062	0.323
HPWS.t2 → Health well-being.t3	0.070	0.026	0.006	0.025	0.150
Health well-being.t2 → Individual performance.t3	0.037	0.004	0.034	0.002	0.121
Health well-being.t3 → Individual performance.t4	0.075	0.040	0.041	0.027	0.163
HPWS.t1 → Health well-being.t2 → Individual performance.t3	0.008	0.003	0.020	0.000	0.020
HPWS.t2 → Health well-being.t3 → Individual performance.t4	0.004	0.002	0.030	0.000	0.032
Age → Individual performance.t3	0.001	0.002	0.681		
Age → Individual performance.t4	0.000	0.002	0.862		
Age → Health well-being.t2	0.007	0.003	0.022		
Age → Health well-being.t3	0.000	0.003	0.912		
Education → Individual performance.t3	0.010	0.033	0.760		
Education → Individual performance.t4	−0.065	0.033	0.046		
Education → Health well-being.t2	0.081	0.041	0.050		
Education → Health well-being.t3	0.079	0.033	0.069		
Gender → Individual performance.t3	−0.064	0.053	0.230		
Gender → Individual performance.t4	−0.060	0.052	0.251		
Gender → Health well-being.t2	0.002	0.066	0.980		
Gender → Health well-being.t3	−0.003	0.055	0.950		

SE, standard error; 95% CI, 95% confidence interval; LCI, lower limit of confidence interval; UCI, upper limit of confidence interval.

## Discussion

This study aimed to shed new light on the underlying mediating processes of the relationship between High-Performance Work Systems (HPWS) and individual performance. In our longitudinal analysis, we initially investigated the impact of perceived HPWS on individual performance. Subsequently, we examined the mediating roles of happiness well-being and health well-being in the link between HPWS and performance.

Our findings indicate that employees’ perceptions of HPWS significantly influence their subsequent performance. Specifically, we observed a positive relationship between HPWS in Wave t and individual performance in subsequent waves. To investigate the relationship between employee well-being and the impact of HPWS on performance, we employed mediation models. These models allowed us to examine the indirect effects of perceived HPWS on individual performance through two mediating factors: happiness well-being and health well-being. By analyzing these mediation models, we were able to assess the influence of HPWS on individual performance in relation to employee well-being. As demonstrated previously, both happiness well-being and health well-being play a significant role in mediating the relationship between perceived High-Performance Work Systems (HPWS) and individual performance. However, it should be noted that the coefficients associated with these mediating variables are relatively small. In our empirical analysis, we employed a latent structural equation modeling approach to test the mediation hypotheses, which may have led to the reduction in the coefficients. Additionally, the direct effects between perceived HPWS and individual performance may also impose constraints on the coefficients of the indirect effects.

The data indicate that happiness well-being positively mediates the relationship between HPWS and individual performance. This finding aligns with the mutual gains perspective commonly found in HR literature (Peccei et al., 2013). Moreover, the data also indicate that health well-being serves as a positive mediator in the relationship between HPWS and individual performance. This finding diverges from our initial hypothesis regarding the negative mediating mechanisms of health-well-being and can potentially be attributed to potential non-linear effects of HRM practices on employee well-being. According to Warr (2007), the negative effects of HR practices on employee health only occur at high level of job characteristics that causes extra job demands. However, a significant proportion of employees seems to fall below this tipping point and perceive HRM practices as beneficial to them (Warr, 2007).

### Theoretical implications

First, this work makes substantial contributions in clarifying the role of various dimensions of well-being as mediators in the relationship between HPWS and individual performance (Peccei and Van De Voorde, 2019). Differing from previous studies that primarily examined a singular aspect of employee well-being (Peccei and Van De Voorde, 2019), our research expanded upon this by investigating happiness and health well-being, within the framework of the JD-R model. The findings of our study demonstrate that both happiness and health well-being play a positive mediating role in the relationship between HPWS and performance. These findings suggest that by improving employees’ happiness and health well-being, organizations can foster individual performance, which aligns with the perspective of mutual gains (Van De Voorde et al., 2012; Peccei et al., 2013). The findings provide additional clarity to the theoretical assumption that there are trade-offs between different dimensions of employee well-being (Ogbonnaya and Messersmith, 2019; Guerci et al., 2022). The findings indicate that the implementation of robust HPWS can simultaneously enhance both happiness and health well-being of the employee.

Second, our study also makes a valuable contribution to the increasing need for rigorous longitudinal research that incorporates temporal aspects. By employing a longitudinal research design, this study takes a step toward examining a dynamic chain between HPWS, well-being, and individual performance. There is a pressing need for a greater emphasis on dynamic relationships within the strategic HRM field. Most existing studies have relied on cross-sectional research designs to explore these inherently dynamic relationships, thus providing preliminary tests of strategic HRM theory (Mitchell and James, 2001; Roe, 2008). By considering temporal effects, this study provides a more accurate and comprehensive test of the assumptions prevalent in the field of HRM.

### Practical implications

Our findings suggest several practical implications. First, the analyses highlight the significance of enhancing employees’ experiences and therefore perceptions of HPWS. Based on the conceptualization in our study, HPWS is most effective when it concurrently enhances employees’ ability, motivation, and opportunity to perform. Although not exhaustive, this list can serve as a valuable starting point for organizations aiming to implement effective HPWS. The HRM literature at the individual level (e.g., Alfes et al., 2013; Kehoe and Wright, 2013; Fu et al., 2018) has shown that perceptions of HRM are more effective than intended or implemented HRM in predicting performance change (Kuvaas and Dysvik, 2010). In practice, there can be discrepancies between the HRM practices that organizations intend to implement and the practices that employees perceive or experience. Due to implementation variability and individual diversity, the links between intended or implemented HRM and perceived HRM tend to be weak (Kuvaas, 2008; Fu et al., 2018). Therefore, organizations should prioritize investing in measures to ensure the communication to align managers implemented HPWS with employee perceptions of it.

A second implication of these findings is that they offer managers a deeper and broader understanding of the benefits of employee well-being. Through enhanced positive perceptions of their jobs and work environments, employees can be happier and more productive. Consistent with “economics of happiness” studies, promoting a psychologically happy workforce is a valuable investment for an organization (Graham, 2005). Furthermore, our analysis indicates the significance of implementing a comprehensive system of health-related HR practices. According to the data, organizations should integrate HR practices into their HPWS that specifically aim to improve the mental well-being of employees. For example, organizations can adapt flexibility into HPWS practices can help reduce harm. For employees, flexibility implies options like working from home, flexible shifts, and staggered shifts, although these do not necessarily reduce the workload. Our research indicates that while implementing HPWS can be beneficial for improving task performance, it is important to recognize that HPWS can also impact overall well-being, which in turn can lead to increased performance.

### Limitations and future research directions

Although our longitudinal and multidimensional research design offers some advantages, this study has limitations. First, our study’s mediation models that address different well-being dimensions allowed us to examine the relationship between HPWS, well-being, and performance better, which is an important step forward compared with previous studies. However, our study did not include conditional factors, such as managers’ behaviors (Den Hartog et al., 2012). As a result, we recognize that other factors may have influenced the relationships we are interested in. An important way of strengthening current understanding is by extending the current mediation analyses to understand better when the hypothesized relationships between HPWS, well-being, and performance are likely to hold. Therefore, future studies are suggested to develop and test more complex moderated-mediation models of the links among HPWS, well-being, and performance (Peccei and Van De Voorde, 2019).

Regarding the measures of HPWS, there is a limitation in the extent to which these practices emphasize resources over demand aspects. Our HPWS measure was developed based on various HPWS practices, which may not equally reflect both job demands and job resources and not provide a balanced representation in terms of evaluating HPWS-related demands and resources in equal detail. Therefore, it would be interesting for future studies to develop the measurement scale of HPWS in a more balanced manner that equally addresses both the resource and demand aspects.

Our research focused specifically on employees in the United Kingdom. While our sample provided a unique opportunity for longitudinal research, it is important to acknowledge that the generalizability of our findings to other countries, particularly in different regions of the world, may be limited. Despite these limitations, the present study offers valuable insights for future research that examines the role of employee well-being in the link between HRM practices and performance. Furthermore, it lays the groundwork for further research investigating the temporal relationships among HRM, well-being, and performance.

## Conclusion

This study explores happiness well-being and health well-being as mechanism through which employees’ perceptions of HPWS influence individual performance. We leveraged a four-wave longitudinal dataset to study two dimensions of well-being in the HPWS-performance relationship. We found that perceived HPWS have positive effects on individual performance over time. We also found that both happiness and health fit the mutual gains perspective and positively mediate the relationship between HRM perceptions and individual performance. Our results shed light on how different dimensions of employee well-being are influenced by HPWS and further affect their individual performance. They uncover nuances of the longitudinal relationship between them that could not be explored in previous cross-sectional studies.

## Data availability statement

Due to the nature of this research, participants of this study did not agree for their data to be shared publicly. Requests to access the datasets should be directed to maria.km@leicester.ac.uk.

## Ethics statement

Ethical approval was not required for the study involving human participants in accordance with the local legislation and institutional requirements. Written informed consent to participate in this study was not required from the participants in accordance with the national legislation and the institutional requirements.

## Author contributions

LS: Conceptualization, Data curation, Formal analysis, Methodology, Writing—original draft, Writing—review and editing. MV: Conceptualization, Methodology, Supervision, Writing—original draft, Writing—review and editing. DK: Conceptualization, Methodology, Validation, Writing—original draft, Writing—review and editing. KV: Conceptualization, Methodology, Validation, Writing—original draft, Writing—review and editing. MK-M: Conceptualization, Data curation, Funding acquisition, Investigation, Methodology, Project administration, Validation, Writing—original draft, Writing—review and editing.
