# Supplementary material for: High-performance work systems and individual performance: a longitudinal study of the differential roles of happiness and health well-being
Source: Front Psychol. 2024 Jan 17;14:1261564. doi: 10.3389/fpsyg.2023.1261564 (PMC10829052; doi:10.3389/fpsyg.2023.1261564)
Supplement: Supplementary file 1 [file Table_1.docx]

Appendix 1 | Structural equation modeling analysis results: individual performance and HPWS.

| **Model** | **c^2^** | **df** | **RMSEA** | **CFI** | **NFI** | **TLI** | **IFI** |
| --- | --- | --- | --- | --- | --- | --- | --- |
|  | **612.76** | **370** | **0.041** | **0.97** | **0.94** | **0.97** | **0.97** |
| **Path** | **Unstandardized coefficient** | | | **SE** | ***P*** | **Standardized coefficient** | |
| Individual performance.t1→HPWS.t2 | 0.055 | | | 0.077 | 0.471 | 0.024 | |
| Individual performance.t2→HPWS.t3 | −0.089 | | | 0.062 | 0.152 | −0.043 | |
| Individual performance.t3→HPWS.t4 | −0.118 | | | 0.066 | 0.073 | −0.051 | |

Appendix 2 | ANOVA test on perceived HPWS, happiness well-being, health well-being and individual performance.

|  | **Perceived HPWS** | | **Individual performance** | | **Happiness well-being** | | **Health well-being** | |
| --- | --- | --- | --- | --- | --- | --- | --- | --- |
| **Organization** | **Mean** | **Std. Dev** | **Mean** | **Std. Dev** | **Mean** | **Std. Dev** | **Mean** | **Std. Dev** |
| Org1 | 4.00 | 0.22 | 4.33 | 0.58 | 4.50 | 0.50 | 4.56 | 0.48 |
| Org2 | 4.01 | 1.15 | 4.29 | 0.95 | 4.23 | 0.86 | 3.94 | 1.10 |
| Org3 | 4.78 | 1.18 | 4.74 | 0.60 | 4.74 | 0.87 | 4.55 | 0.76 |
| Org4 | 3.67 | 1.55 | 4.40 | 0.43 | 4.17 | 0.77 | 3.37 | 1.05 |
| Org5 | 4.67 | 1.45 | 4.67 | 0.58 | 5.11 | 0.42 | 4.72 | 0.67 |
| Org6 | 4.32 | 1.15 | 4.39 | 0.56 | 4.06 | 1.12 | 4.03 | 0.93 |
| Org7 | 4.31 | 0.98 | 4.46 | 0.50 | 3.81 | 0.95 | 3.48 | 0.99 |
| Org8 | 5.50 | 0.00 | 5.00 | 0.00 | 4.00 | 0.00 | 4.83 | 0.00 |
| Org9 | 3.32 | 1.47 | 4.43 | 0.54 | 4.07 | 0.92 | 3.71 | 0.95 |
| Org10 | 4.09 | 1.34 | 4.45 | 0.53 | 4.22 | 1.10 | 4.07 | 1.08 |
| Org11 | 5.04 | 0.18 | 3.83 | 0.24 | 4.17 | 1.41 | 3.00 | 0.94 |
| Org12 | 4.26 | 1.24 | 4.55 | 0.74 | 3.54 | 1.24 | 3.14 | 1.21 |
| Org13 | 3.47 | 1.18 | 4.53 | 0.50 | 4.00 | 0.56 | 3.72 | 0.69 |
| Org14 | 3.72 | 1.08 | 4.07 | 0.60 | 4.17 | 0.57 | 4.10 | 0.58 |
| Org15 | 4.12 | 1.26 | 4.49 | 0.61 | 4.06 | 1.07 | 3.71 | 1.14 |
| Org16 | 4.47 | 1.28 | 4.76 | 0.42 | 4.74 | 0.97 | 4.27 | 1.12 |
| Total | 4.11 | 1.27 | 4.47 | 0.63 | 4.08 | 1.07 | 3.78 | 1.11 |
| Bartlett’s test for equal variances | | | | | | | | |
| chi2(14) | 11.31 | | 22.44 | | 17.21 | | 12.97 | |
| Prob>chi2 | 0.66 | | 0.07 | | 0.25 | | 0.52 | |
